# Towards Rational Use of Antibiotics for Suspected Secondary Infections in Buruli Ulcer Patients

**DOI:** 10.1371/journal.pntd.0002010

**Published:** 2013-01-24

**Authors:** Yves T. Barogui, Sandor Klis, Honoré Sourou Bankolé, Ghislain E. Sopoh, Solomon Mamo, Lamine Baba-Moussa, Willem L. Manson, Roch Christian Johnson, Tjip S. van der Werf, Ymkje Stienstra

**Affiliations:** 1 Programme National de Lutte Contre la Lèpre et l'Ulcère de Buruli, Ministère de la Santé, Cotonou, Bénin; 2 University of Groningen, University Medical Center Groningen, Department of Internal Medicine/Infectious Diseases, Groningen, The Netherlands; 3 Département de Génie de Biologie Humaine, Ecole polytechnique de l'Université d'Abomey-Calavi, Cotonou, Bénin; 4 Agogo Presbyterian Hospital, Department of Surgery, Agogo, Ghana; 5 Laboratoire de biologie et de typage moléculaire en microbiologie, département de biochimie et biologie cellulaire, Faculté des sciences et techniques, Université d'Abomey-Calavi, Cotonou, Bénin; 6 University of Groningen, University Medical Center Groningen, Department of Medical Microbiology, Groningen, The Netherlands; 7 Fondation Raoul Follereau, Cotonou, Bénin; University of Tennessee, United States of America

## Abstract

**Background:**

The emerging disease Buruli ulcer is treated with streptomycin and rifampicin and surgery if necessary. Frequently other antibiotics are used during treatment.

**Methods/Principal Findings:**

Information on prescribing behavior of antibiotics for suspected secondary infections and for prophylactic use was collected retrospectively. Of 185 patients that started treatment for Buruli ulcer in different centers in Ghana and Bénin 51 were admitted. Forty of these 51 admitted patients (78%) received at least one course of antibiotics other than streptomycin and rifampicin during their hospital stay. The median number (IQR) of antibiotic courses for admitted patients was 2 (1, 5). Only twelve patients received antibiotics for a suspected secondary infection, all other courses were prescribed as prophylaxis of secondary infections extended till 10 days on average after excision, debridement or skin grafting. Antibiotic regimens varied considerably per indication. In another group of BU patients in two centers in Bénin , superficial wound cultures were performed. These cultures from superficial swabs represented bacteria to be expected from a chronic wound, but 13 of the 34 (38%) *S. aureus* were MRSA.

**Conclusions/Significance:**

A guide for rational antibiotic treatment for suspected secondary infections or prophylaxis is needed. Adherence to the guideline proposed in this article may reduce and tailor antibiotic use other than streptomycin and rifampicin in Buruli ulcer patients. It may save costs, reduce toxicity and limit development of further antimicrobial resistance. This topic should be included in general protocols on the management of Buruli ulcer.

## Author Summary

Buruli ulcer (BU) is a neglected, emerging disease caused by *Mycobacterium ulcerans*. BU usually starts as a nodule, papule, plaque, or oedema. When left alone, the lesion breaks open and a typical painless ulcer with undermined edges appears which can progress to a large necrotic lesion. BU is treated with antibiotics (streptomycin and rifampicin) and surgery if necessary. Apart from these two antibiotics, patients frequently receive other antibiotics during treatment. In files from patients treated in Benin and Ghana we found that in admitted patients a median of two antibiotic courses were prescribed. Only twelve patients received antibiotics for a suspected secondary infection, all other courses were prescribed as prophylaxis of secondary infection extended till 10 days on average after excision, debridement or skin grafting. In another patient group in Benin, superficial wound swabs from Buruli ulcers were performed and showed a high rate of MRSA. We propose a guideline for rational antibiotic treatment for suspected secondary infections or prophylaxis. Adherence to the proposed guideline will have a major impact on antibiotic use other than streptomycin and rifampicin in Buruli ulcer patients, saving costs, toxicity and development of antimicrobial resistance.

## Introduction

Buruli ulcer (BU) is a neglected, emerging disease caused by *Mycobacterium ulcerans*. BU usually starts as a nodule, papule, plaque, or oedema. When left alone, the lesion breaks open and a typical painless ulcer with undermined edges appears which can progress to a large necrotic lesion. Sometimes the bone can be affected and amputation may be necessary. Until 2004, the only available treatment was surgical removal of affected tissue. Since 2004, streptomycin and rifampicin have been used to treat BU [1]–[3].

Secondary infection is often thought to be responsible of severe complications in BU [4]–[6]. In chronic diabetic foot ulcers and thermal burn wounds, secondary infections increase time to healing and prolong hospital stay [7]–[10]. The Infectious Diseases Society of America (IDSA) guidelines on diabetic foot infections states that infection should be diagnosed clinically on the basis of the presence of purulent secretions (pus) or at least 2 of the cardinal manifestations of inflammation (redness, warmth, swelling or induration, and pain or tenderness) [11], [12].

Although the incidence of secondary infections in BU is unknown, antibiotics may be frequently prescribed for this indication. It is equally unknown which bacteria these antibiotics should target and what the susceptibility of these bacteria is. Furthermore, the prescribing behaviour of antibiotics used as prophylaxis after surgery or skin grafting is unknown. Only limited data are available on resistance of microbes in Bénin and Ghana; a study in Ghana found that 18% of the *S. aureus* were MRSA [12]. The few studies published on the prevalence of MRSA in Bénin, showed percentages varying from 17–36% depending on the type of samples studied [13], [14]. Resistance patterns of *E. coli* were described in faeces from healthy volunteers and patients with diarrhea. No Extended-Spectrum beta-lactamase (ESBL) producing enterobacteriaceae were found in these two studies, but there was a high resistance to locally used antibiotics [15], [16]. In a 500 bed hospital in Abomey 22% of the *E. coli* were ESBL positive [17]. An antibiotic policy that is adjusted to the expected microbes and resistance of these microbes can make antibiotic use more effective, with lower daily defined doses of antibiotics per person, leading to less side-effects, and with less resistance. Such policy will also save resources in an environment where resources are scarce.

The prescribing behaviour of antibiotics for secondary infections or as prophylaxis in BU interventions was studied, and cultures of ulcers were taken to provide data for the development of future guidelines for the use of antibiotics for this indication.

## Methods

### Prescribing behaviour

Data were retrieved from files of patients that started treatment with streptomycin and rifampicin in the period August–October 2009 in the ‘centre de dépistage et de traitement de l'ulcère de Buruli’, Lalo, Bénin. The same data were retrieved from hospital files on patients that started treatment in the period August–December 2009 in the ‘centre dépistage et de traitement de l'ulcère de Buruli’, Allada, Bénin, and in the period March 2008–March 2009 in Agogo Presbyterian Hospital, Agogo, Ghana. Records were studied in August and September 2010, so that follow-up of these patients was already completed. Patient characteristics and the type of lesion were recorded. Data on antibiotic use different from rifampicin/streptomycin, as well as the indication for these prescriptions, the dosage and duration as well as the clinical presentation at the start of treatment were retrieved.

### Microbiological methods

Between October and December 2010, 20 consecutive patients with an ulcerative lesion reporting for BU treatment were enrolled after consent and followed longitudinally. Before the start of treatment, and before washing or the application of antiseptics, a swab was taken both from the undermined edge and from the center of the ulcer. Swabs were also taken at 6 weeks after start of treatment and at 12 weeks (this is 4 weeks after finishing the 8 weeks rifampicin/streptomycin). Apart from these 20 patients followed in time, we intended to enrol 25 patients in the villages and 25 patients admitted in the hospitals after consent with only one culture taken at a random moment during or after treatment. The clinical presentation (including local and/or systemic signs of a secondary infection) and previous use of antibiotics was recorded for all patients.

Results of the cultures were not reported to the treating physicians in order to avoid interference with antibiotic prescribing that would be based on superficial cultures.

Samples were cultured on a medium of Trypcase Soy Agar+5% Sheep Blood and Chapman Agar (both Biomerieux EMB agar). The different microbial isolates were differentiated and antimicrobial susceptibility was performed on Müller Hinton agar with antibiotic discs (Rosco Diagnostica). Detection of methicillin resistance was done on Műller Hinton agar with the addition of 5% NaCl. The results of the antibiograms were reported as either susceptible, intermediate resistance, or resistant. Apart from the antibiotics tested in the routine setting, rifampicin and clarithromycin discs were added to the antibiograms of gram positive microbes, and streptomycin discs were added to antibiograms of both gram positive and negative microbes. Cultures and antibiotic susceptibility testing were performed at the National Public health Laboratory, Cotonou. The method used for susceptibility testing was the agar medium diffusion method (Kirby Bauer method). The internal quality control is done with the following reference strains:*E. coli* ATCC25922 and *S. aureus* ATCC25923. The laboratory participates in international quality control with the following organizations: Institute Pasteur in Paris, the WHO Collaborating Centers Faro in Marseilles (France), and MDSC in Ouagadougou, Burkina Faso.

### Statistical methods

From the 20 patients with cultures taken at the same time from the center and the border of the ulcer, only the results of the border of the ulcer were used for the descriptive analysis.

### Ethics

The protocol and consent forms of the study were approved by the ethical review committee of the Ministry of Health (Direction de la Formation et de la Récherche en Santé, nr IRB6860). For participants in the part of the study obtaining swabs from the Buruli ulcer lesions, written and verbal informed consent or assent was obtained from all participants aged 12 years or older, and consent from parents, care takers, or legal representatives of participants aged between 12 and 18 years of age.

## Results

### Prescribing behavior

In total, 185 patients started treatment with streptomycin and rifampicin in the study periods. 147 Patients had an ulcer, 38 had a plaque as the only lesion. Four patients (2.7%) had both an ulcer and a nodule. Median age was 12 years old. Two patients were known to be HIV positive; the other patients did not have a relevant medical history.

Of these 185 patients, 51 were admitted because of the severity of the disease or because of distance to health care center. Of the 51 admitted patients, 40 (78%) received at least one course of antibiotics other than streptomycin and rifampicin during their admission. The median number (IQR) of antibiotic courses for admitted patients was 2 (1, 5), with a maximum number of courses of 13. In Table 1, the different antibiotic strategies for suspected secondary infections and prophylaxis extended after three different surgical interventions are presented. Apart from the antibiotics prescribed in Table 1, two patients received treatment for suspected sepsis with a secondarily infected BU lesion as focus. One of them received ampicillin, gentamicin and metronidazole and the other received ceftriaxone and metronidazole. Another patient received ampicillin and metronidazole as prophylaxis extended after bone surgery for disseminated BU.

**Table 1:** Antibiotic treatment given to patients for different Buruli ulcer related indications.

INDICATION (IN 51 ADMITTED PATIENTS)	SECONDARY INFECTION (N = 12)				PROPHYLAXIS EXTENDED AFTER EXCISION (N = 19)				PROPHYLAXIS EXTENDED AFTER SKIN GRAFTING (N = 37)				PROPHYLAXIS EXTENDED AFTER DEBRIDEMENT (N = 5)			
	Country A	Country B	Total	Mean Duration (days)	Country A	Country B	Total	Mean duration (days)	Country A	Country B	Total	Mean duration (days)	Country A	Country B	Total	Mean Duration (days)
Flucloxacillin	1	0	1	7	0	3	3	10	0	9	9	10	1	1	2	9
Ampicillin/amoxicillin	0	1	1	5	0	5	5	8	0	3	3	7	-	-	-	-
Ciprofloxacin	0	1	1	10	-	-	-	-	0	3	3	10	-	-	-	-
Gentamicin	1	0	1	5	-	-	-	-	-	-	-	-	1	0	1	5
Cotrimoxazole	-	-	-	-	-	-	-	-				-	0	1	1	10
Penicillin and flucloxacillin	1	0	1	5	-	-	-	-	-	-	-	-	-	-	-	-
Flucloxacillin and metronidazole	0	1	1	10	0	6	6	10	0	3	3	unknown	-	-	-	-
Flucloxacillin and gentamicin	-	-	-	-	-	-	-	-	11	0	11	F7,G5	-	-	-	-
Amoxicillin and metronidazole	0	1	1	3	0	1	1	10	0	4	4	10	-	-	-	-
Ciprofloxacin and metronidazole	0	3	3	9	0	4	4	10	0	2	2	10	0	1	1	C7,M5
Clindamycin and gentamicin	1	0	1	10	-	-	-	-	1	0	1	5	-	-	-	-
Ceftriaxone, gentamicin and metronidazole	0	1	1	C10, G5, M5	-	-	-	-	-	-	-	-	-	-	-	-
Penicillin G, flucloxacillin and gentamicin	-	-	-	-	-	-	-	-	1	0	1	P3, F10, G5	-	-	-	-

Different antibiotic combinations were started during the treatment with streptomycin and rifampicin sixteen times. Median number of days passed between start of streptomycin and rifampicin and the first time other antibiotics were prescribed was 63 days.

The clinical signs reported when starting a course of antibiotics to treat a Buruli ulcer related infection were diverse (Table 2).This prescribing behaviour resulted in a high number of antibiotics prescribed per 100 patient days of hospitalization (Table 3).

**Table 2:** Clinical signs reported before start of antibiotics for a suspected secondary infected BU.

OOZING, PUS, ‘WOUND LOOKS DIRTY’	3
Raised WBC or fever	2
Fever with a local sign (swelling, warmth, tenderness)	2
Warmth, swelling or tenderness	2
Oozing and swelling	1
Unknown	1
Necrotic tissue	1

WBC = White Blood cell Count.

**Table 3:** Number of days of prescribed antibiotics per 100 days in hospital.

ANTIBIOTICS*	DAYS ANTIBIOTICS USED IN THERAPEUTIC DOSAGE PER 100 DAYS IN HOSPITAL.
Penicillin G	0.1
Cloxacillin/Flucloxacillin	6.2
Amoxicillin/Ampicillin	2.9
Ceftriaxone	0.4
Ciprofloxacin	3.2
Gentamicin	1.9
Cotrimoxazole	1.1
Metronidazole	6.9
Clindamycin	0.4
***Total apart from SR***	**23.0**

* Standard treatment of *M. ulcerans* with 56 days of rifampicin and streptomycin not included in this table.

### Microbiological results of Buruli ulcer lesions in Bénin

#### Culture results

In 71 patients (20 patients with follow-up cultures, 26 hospitalized patients and 25 outpatients) 106 cultures were taken from a BU lesion. *S. aureus* was less frequently found during therapy with streptomycin and rifampicin, further distribution (Table 4) of results seemed similar before, during or after antibiotic therapy. Looking separately at the 20 patients that were followed longitudinally (before the start of antibiotics, after 6 and after 12 weeks), showed a similar pattern with less frequently *S. aureus* found in the culture after 6 weeks of antibiotic therapy. In these 20 patients cultures were taken from the center of the lesion, and from the undermined edges of the ulcers before the start of treatment. Isolates did not significantly differ between these two, but cultures from the undermined edges were more often negative than from the center of the ulcer (18% vs. 9%, p = 0.34 Chi-square). None of the cultures was taken during antibiotic treatment other than streptomycin or rifampicin.

**Table 4:** Results of cultures in 71 patients before, during or after treatment with streptomycin and rifampicin.

	before SR (59cultures)	during SR (16cultures)	after SR (31cultures)	Total (106 cultures)
Group A streptococcus	4	0	2	**6**
Group B or C streptococcus	2	1	0	**3**
*S. aureus*	15	0	11	**26**
*S. epidermidis*	0	1	0	**1**
Staphylococcus sp	0	0	1	**1**
*Pseudomonas aeruginosa*	13	5	12	**30**
*Enterobacteriaceae*	19	6	8	**33**
*Negative culture*	19 (32%)	4(25%)	7(23%)	**30(28%)**

For patients followed longitudinally, only cultures from the center of the ulcer were included at inclusion (cultures from border gave similar results).

#### Clinical presentation at moment of culture

At the moment the culture was performed, 38 patients had one local sign of a secondary infection and 12 patients had two local signs of a secondary infection. Of these 12 patients with two local symptoms, only one culture turned out to be negative. Only one of these 12 patients was clinically considered to have a secondary infection and started treatment with additional antibiotics after the cultures were taken. In the other positive cultures, a distribution of bacteria similar to Table 4 was found. At the moment cultures were taken none of the patients showed a systemic sign of a secondary infection.

#### Susceptibility pattern of bacteria cultured

In total 124 microorganisms were cultured, including the follow-up cultures and the cultures at the border and at the center of the lesion. The susceptibility pattern of the cultured microorganisms is presented in Table 5, susceptibility of the cultured MRSA in Table 6. Thirteen of the 34 (38%) *S. aureus* isolates were resistant to oxacillin and were therefore probably MRSA. This percentage was not substantially different in patients before admission or before start of antibiotic treatment. Based on resistance to ceftriaxone and susceptibility to amoxicillin/clavulanic acid, we expect that there was one ESBL producing *Klebsiella pneumoniae*.

**Table 5:** Susceptibility of cultured organisms (other than *M. ulcerans*) in Buruli ulcer lesions in Benin.

	*S. aureus*, n = 34			coagulase negative Staphylococcus, n = 2			beta hemolytic Streptococcus, n = 14			*Pseudomonas aeruginosa*, n = 33			Enterobacteriaceae, n = 41		
	S	I	R	S	I	R	S	I	R	S	I	R	S	I	R
Amoxicillin							12	0	2						
Amoxicillin/clavulanic acid													11	5	22
Oxacillin	21	0	13	0	0	2									
Cefoxitin															
Ceftriaxone										13	14	6	26	3	8
Cefuroxime													0	1	2
Gentamicin	34	0	0	2	0	0				26	0	7	34	0	5
Streptomycin	7	6	20	0	0	2				6	2	17	6	8	22
Clarithromycin	21	2	11	0	0	2	9	3	2						
Chloramphenicol	21	0	13	0	0	2	7	1	6				12	3	23
Nalidixic acid										2	1	16	11	2	6
Ofloxacin	31	3	0	1	0	1	11	1	2	31	0	0	30	1	7
Rifampicin	25	1	8	0	0	2	9	2	3						

Cultures taken from both the center and the border of the ulcer at start of treatment in patients followed longitudinally were included, leading to a different number of cultures than in Table 4.

**Table 6:** Sensitivity of the cultured MRSA.

MRSA = 13	S	I	R
Gentamicin	13	0	0
Streptomycin	1	2	10
Clarithromycin	4	1	8
Chloramphenicol	4	0	9
Ofloxacin	10	3	0

## Discussion

Currently there is no specific guideline for the prescription of additional antibiotics to BU patients suspected to have secondary infection. However, our findings indicate that prescribing additional antibiotics is widespread among BU patients that are admitted to the hospital because of the severity of their BU or distance to health care. Moreover, the type and duration of these antibiotic courses is highly variable, even within the same indication. Antibiotics were most frequently prescribed as prophylaxis of secondary infections extended after surgical procedures rather than for the treatment of suspected secondary infections. Surprisingly, duration of this prophylaxis was even longer than for actual treatment of secondary infections. This frequent use of antibiotics leads to unnecessary costs, antibiotic resistance, and side-effects for the patients with possible long term consequences. For example, prescribing gentamicin during the therapy with rifampicin and streptomycin imposes a serious risk of aminoglycoside toxicity.

In general, skin surgery is not an indication for antimicrobial prophylaxis, therefore prophylaxis after excision and after debridement [18] is not considered as indicated. However, there is some inconclusive evidence that in skin grafts systemic perioperative antibiotic prophylaxis contributes to the autograft survival [19]. Ramos et al. found a rate of autograft survival for the group of patients with burns using two days of perioperative antibiotic prophylaxis of 97% versus 87% in the group without prophylaxis [20]. In patients with arterial and venous ulcers no differences in graft survival was observed with perioperative use of antibiotics [21].

**Figure 1:**
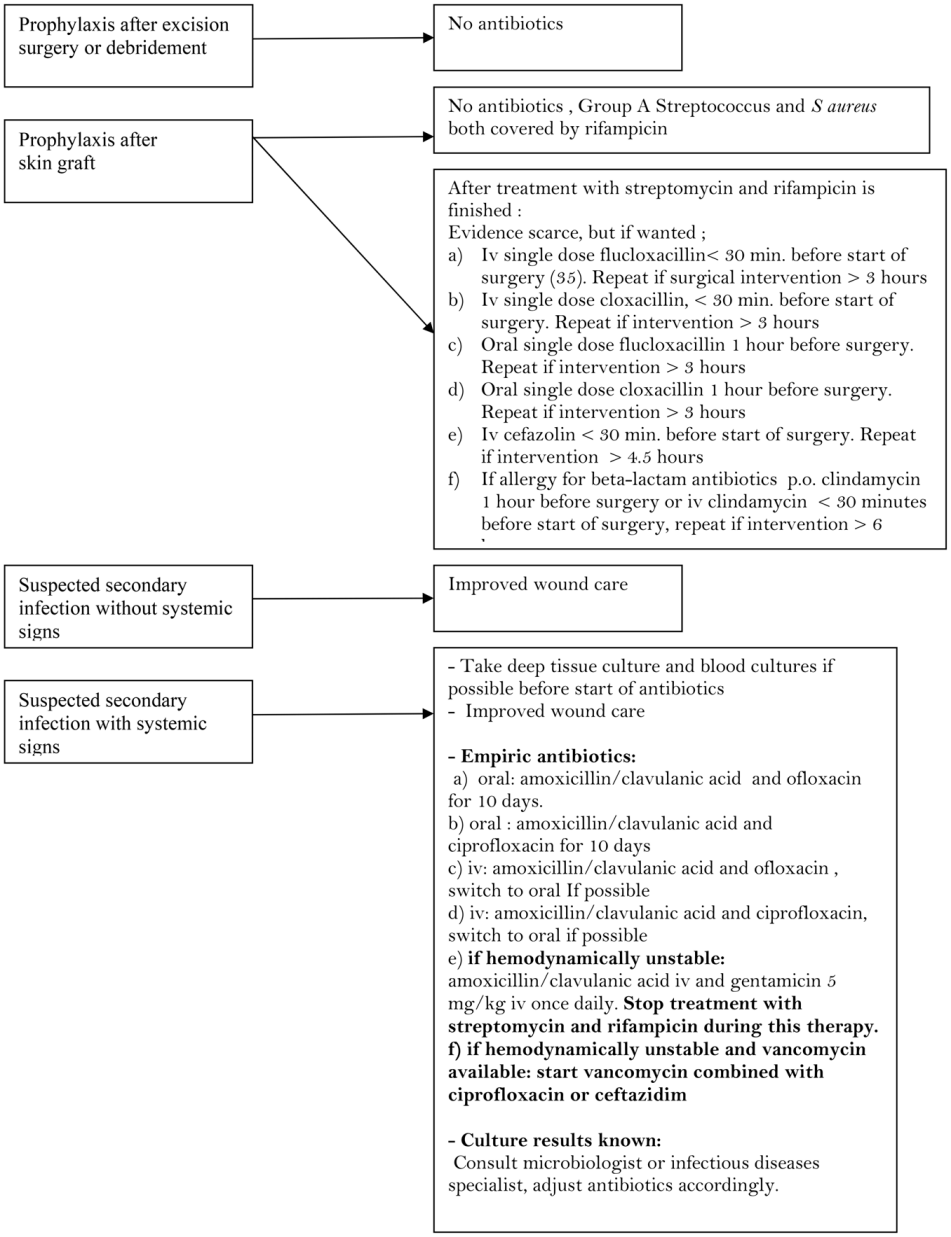
Suggested guideline for use of antibiotics in prophylaxis and suspected secondary infections. * Ciprofloxacin is not generally recommended for use in children, but appears relatively safe [36].

As stated by the IDSA guidelines it is unlikely that benefit is conferred by the administration of additional doses after the patient has left the operating room [18], [22], [23]. Antimicrobial prophylaxis should certainly be discontinued within 24 hours of the operative procedure since prolonged antibiotic prophylaxis contributes to antimicrobial resistance [18], [24], [25]. In case clinicians decide to give perioperative antibiotics in skin grafting, advice for use of appropriate antibiotics is given in Figure 1. Although MRSA is frequent and community acquired, suggested antibiotic therapy as prophylaxis in skin grafting in Figure 1 does not cover MRSA. The alternatives vancomycin or ofloxacin/ciprofloxacin are not appropriate as prophylaxis due to resistance development to these antibiotics and/or costs. Studies on the use of vancomyin as prophylaxis in medical centers with high MRSA prevalence are controversial in preventing surgical site infections [26], [27].

Report of clinical symptoms of secondary infections may not always have been complete in the files, but different clinical signs were reported in patients receiving antibiotics for suspected secondary infection. These clinical signs seem nonspecific; in patients not clinically suspected to have a secondary infection, the same local and systemic signs were frequent as well. The diagnosis of a secondary infection of the BU therefore remains difficult to ascertain. A paradoxical response may be an alternative diagnosis in patients suspected of a secondary infection [28]. A superficial culture of the wound as done in the study is certainly not helpful in the diagnosis of a secondary infection and can not guide individual care [29], [30], [31]. Superficial cultures as done in this study, can not guide individual care. In this study, we performed cultures to make an inventory of isolates and local resistance patterns to enable the first steps to an antibiotic guideline. Superficial swabs yield a greater range of organisms than do deeper tissue material due to contaminants not involved in the secondary infection and yet may fail to identify some of the deep-seated organisms. If a culture is needed to guide antibiotic therapy, tissue specimens obtained by biopsy, ulcer curettage or aspiration are preferred [11]. Such cultures would have been preferable for this study but were not performed for ethical reasons. Such procedures are more invasive, and moreover, most included patients were not suspected to have a secondary infection and they would therefore not benefit from a deep tissue biopsy.

Another limitation of the study is the limited number of positive cultures, yet we think this number of isolates reflects the micro-organisms in the superficial swabs in general and this information guides antibiotic treatment suggestion. Further studies are needed to gather more information on the susceptibility of the highly prevalent MRSA. Patients treated in the out-patient setting did not have reported use of antibiotics apart from streptomycin and rifampicin, but we are not informed on antibiotics that may have been used by these patients prescribed by other doctors than the doctors treating their BU or that patients bought without prescription.

The high percentage of MRSA cultured is worrisome. The prevalence of MRSA was equally high among patients before start of treatment, suggesting this MRSA may be community acquired. In Benin, antibiotics are freely available, and are often used without prescription, and a recent study showed that BU patients often use left-over antibiotics to reduce pain and inflammation before reporting to the hospital [32]. Improved wound care and antibiotic therapies in case of clinical suspicion of a secondary infection of the BU are suggested in Figure 1. This figure is based on availability, WHO Essential Drug Lists 2011, costs and the high prevalence of community acquired MRSA.

If treatment contains gentamicin, treatment with streptomycin and rifampicin should be stopped temporarily, to limit toxicity. Dosage of different antibiotics are given in Text S1.

Even though secondary infections were not frequent, the high prevalence of MRSA complicates treatment. Further studies are needed to have a more precise susceptibility pattern of *S. aureus* in this patient population. To deal with the currently found high prevalence of MRSA, facilities at the BU treating centers will have to be improved. Laboratory facilities that enable cultures and testing for MRSA are highly necessary along with knowledge about rational prescribing and the possibility to consult clinical microbiologists or infectious diseases specialist to help interpreting the results. In case patients are suspected to have a secondary infection not responsive to appropriate wound care, a deep tissue biopsy should be sent for culture before the start of antibiotic treatment. In case of systemic signs, blood cultures should also be performed if possible. Vancomycin is on the complementary list of the WHO Essential drug list 2011but it is not available in most centers. However, with the MRSA prevalence as found, it seems an essential drug. Its use is complicated by high costs and need for plasma drug concentration monitoring to limit toxicity.

Treatment of osteomyelitis is not included in the figure; in the year 2000 a study showed that only in 16% of the osteomyelitis patients another germ than *M. ulcerans* was involved [33] Whether this percentage is still accurate is unknown and this should be studied during ongoing drug studies. Especially because of long treatment duration, in case of osteomyelitis, cultures should be sent from bone debridement/biopsy to guide antibiotic therapy for potential non-MU organisms, if possible.

Strategies to optimize wound care in Buruli ulcer patients should be studied, as no information is currently available. However, all measures to prevent healthcare associated infections should be actively implemented in all facilities implementing wound care.

Periodic routine culturing of wounds should be performed (e.g. once every 2 years) to remain updated on the prevalence of MRSA and possible development of resistance. Rational antibiotic prescribing behaviour should be stimulated in centers treating BU [34], [35] Adherence to Figure 1 in prescribing antibiotics different from the streptomycin and rifampicin will have a major impact on antibiotic use in the BU treatment centers, saving money and toxicity and limiting of further antimicrobial resistance development. This topic should be included in general protocols on the management of BU.

## Supporting Information

Text S1 **Dosage of antibiotics in** **Figure 1** **.**
(DOC)

Checklist S1 **STROBE.**
(DOC)
