# Supplementary material for: Towards Rational Use of Antibiotics for Suspected Secondary Infections in Buruli Ulcer Patients
Source: PLoS Negl Trop Dis. 2013 Jan 24;7(1):e2010. doi: 10.1371/journal.pntd.0002010 (PMC3554522; doi:10.1371/journal.pntd.0002010)
Supplement: Text S1 — Dosage of antibiotics in Figure 1 . (DOC) [file pntd.0002010.s001.doc]

**Supporting text S1 – Antibiotic dosage**

Advised dosage of antibiotics in figure 1, if necessary adjust based on kidney/liver function.

Amoxicillin/clavulanic acid:

oral treatment in adults: 500/125mg three times daily, iv: 1000/250 mg three times daily

children: 20/5 mg /kg three times daily

Cefazolin as prophylaxis in aduts: single dose 1000 mg , in children: 50 mg/kg (not more than 1000 mg)

Clindamycin as prophylaxis in adults 600 mg , in children 30 mg/kg (not more than 600 mg)

Cloxacillin as prophylaxis in adults: single dose 1000 mg, in children: 50 mg/kg (not more than 1000 mg)

Ciprofloxacin: adults: 500 mg twice daily, children: 15 mg/kg twice daily

Flucloxacillin as prophylaxis in adults; single dose , 1000 mg, in children 50 mg/kg (not more than 1000 mg)

Gentamicin: 5 mg/kg iv once a day.

Ofloxacin: adults: 200 mg twice daily, children: 15 mg/kg twice daily
